# Chitosan–*Sargassum fusiforme* Polyphenol Edible Films: From Physicochemical and Bioactive Characterization to Shelf-Life Extension of Refrigerated Golden Pompano Fillets

**DOI:** 10.3390/foods15203688

**Published:** 2026-10-16

**Authors:** Le Li, Zhiqing Liu, Siting Wang, Quanhong Li

**Affiliations:** 1 School of Environmental and Quality Testing, Chongqing Chemical Industry Vocational College, No. 2009, East Bodhi Road, Changshou District, Chongqing 401228, China; 2 College of Food Science & Nutritional Engineering, China Agricultural University, No. 17 Tsinghua Dong Road, Beijing 100083, China; 3 School of Food Science and Engineering, South China University of Technology, No. 381 Wushan Road, Guangzhou 510640, China

**Keywords:** *Sargassum fusiforme* polyphenols, chitosan-based edible films, active food packaging systems

## Abstract

The practical application of active food packaging materials is increasingly constrained by performance degradation and functional oversimplification. Although *Sargassum fusiforme* polyphenols (SP) possess potent antioxidant activity, their potential in edible films remains unexplored. In this study, the effects of incorporating varying proportions of SP (0.5–2.0%, *w*/*v*) into Chitosan–*Sargassum fusiforme* polyphenol edible films (CSFFs) on the physicochemical properties and preservative efficacy for chilled golden pompano fillets were investigated. SP incorporation was found to alter film structure and properties significantly. At SP concentrations ≥ 1.0%, reductions in tensile strength, elongation at break, and water vapor permeability were observed, along with increased opacity and enhanced UV-Vis light barrier capacity. SP concentration-dependent increases in DPPH and ABTS radical scavenging activities were revealed, and the CSFFs exhibited broad-spectrum antibacterial activity against six pathogenic strains. In preservation trials at 4 °C, the 2.0% SP coating yielded optimal results, with total bacterial count (5.63 log CFU/g) and total volatile basic nitrogen (18.45 mg/100 g) remaining below acceptable limits on day 15. Additionally, pH increase was inhibited, weight loss was reduced, and declines in hardness and springiness were prevented. Collectively, this study demonstrates that SP can serve as an effective functional component to enhance chitosan-based edible films, offering a scalable active packaging strategy for refrigerated aquatic product preservation.

## 1. Introduction

In recent decades, plastics derived from petrochemical feedstocks have become ubiquitous in food packaging, largely because of their superior mechanical performance and affordability. Nonetheless, the recalcitrant nature of these materials has resulted in substantial environmental accumulation and posed potential risks to food safety [1]. This has prompted the food industry to investigate biopolymer-based packaging systems as viable substitutes, with the dual aim of preserving food quality and mitigating ecological impact. To date, the biopolymers most frequently utilized in food packaging applications comprise chitosan (Ch), cellulose, starch, gelatin, sodium alginate, and whey protein [1]. Of these, Ch—a deacetylated form of the naturally occurring polysaccharide chitin—has found extensive use in the formulation of edible coatings for food preservation, owing to its biodegradability, biocompatibility, non-toxicity, antimicrobial efficacy, and strong film-forming ability [2,3].

The antimicrobial mechanism of Ch is largely ascribed to electrostatic attraction between its protonated amino moieties (–NH_3_^+^) and anionic components present on the surface of bacterial membranes [2]. Nevertheless, the limited water solubility (WS) and modest antimicrobial potency of Ch have constrained its broader utilization in food-related applications. Over the past ten years, antimicrobial edible composite films have been successfully fabricated by introducing natural bioactive agents (such as polyphenols) into the Ch matrix [3,4,5]. Relative to pristine Ch films, these composite formulations display enhanced antimicrobial activity and improved physicochemical characteristics, and have therefore garnered growing interest as food packaging candidates [4,5].

Polyphenols are a class of secondary metabolites widely present in plants, characterized by chemical structures containing one or more aromatic rings with multiple hydroxyl substituents. Common polyphenolic compounds include phenolic acids, flavonoids, tannins, and stilbenes [6]. Compared with synthetic preservatives, natural polyphenols offer higher safety profiles [4,5]. Numerous studies have confirmed that polyphenols exhibit excellent antibacterial activity against spoilage microorganisms, primarily attributed to their ability to disrupt microbial cell membrane integrity, inhibit key intracellular enzyme activities, and induce oxidative stress responses [7,8]. However, most natural polyphenols suffer from poor chemical stability and are susceptible to oxidation or degradation induced by light, heat, and pH, thereby limiting their direct application in food systems [5]. Therefore, loading polyphenols onto suitable carriers to enhance their stability is an effective strategy. Incorporating polyphenols into the Ch matrix not only enhances the antibacterial and antioxidant properties of the composite film through synergistic effects but also improves the mechanical and barrier properties of Ch films, achieving functional complementarity [6,9].

*Sargassum fusiforme* (*S. fusiforme*) is a common brown alga belonging to the family *Sargassaceae*, mainly distributed in the coastal waters of East Asian countries, including China, Japan, and South Korea [10]. Polyphenols extracted from *S. fusiforme* are natural phenolic compounds that mainly include phloroglucinol derivatives, flavonoids, and phenolic acids [10]. Existing studies have reported that *S. fusiforme* polyphenols (SP) possess good antioxidant, antibacterial, and anti-inflammatory activities [10]. The fuhalol-type phlorotannins in SP contain pyrogallol moieties, which efficiently neutralize free radicals via hydrogen atom transfer (HAT) or single-electron transfer (SET) mechanisms, conferring unique antioxidant advantages [6,11]. *S. fusiforme* extracts have also been investigated as natural antimicrobial and antioxidant agents in food products and incorporated into films or coatings based on polymers such as alginate, gelatin, starch, and pectin. However, most *S. fusiforme* resources are still directed towards primary processing or direct consumption, and their polyphenolic components remain underexploited for higher-value applications [6,9,11,12]. In particular, the relationship between the pyrogallol-rich architecture of SP and chitosan film performance, including structural compactness, water vapour barrier, mechanical properties, and bioactivity, has not been systematically elucidated. This study therefore addresses this gap by developing SP-incorporated chitosan films and clarifying the underlying structure–property–function relationships.

Golden pompano (*Trachinotus blochii*), commonly known as golden pomfret, is an important commercially farmed marine fish species widely distributed along the southeastern coast of China and in Southeast Asia [4]. Owing to its tender meat, rich nutrition, and few intramuscular bones, it is highly favored by consumers. However, golden pompano is highly susceptible to microbial contamination and lipid oxidation during storage and transportation, leading to quality deterioration and shortened shelf life, which severely compromises its economic value [13,14]. With increasing consumer demands for food safety and quality, the development of efficient, safe, and green preservation technologies for aquatic products has become an urgent priority. Currently, the preservation of aquatic products mainly relies on technologies such as low-temperature storage, ice storage, partial freezing storage, modified atmosphere packaging, and their combinations [4,5]. However, some of these technologies suffer from high energy consumption and complex equipment requirements, while the use of synthetic preservatives is often associated with potential health risks [4]. In contrast, edible coating preservation technologies based on natural preservatives have attracted increasing attention due to their advantages of safety, environmental friendliness, simple operation, low equipment requirements, and energy efficiency [2,15,16]. Beyond chitosan, scientific investigations have explored alternative active coating materials for fish fillet preservation. Sodium alginate-based coatings incorporated with natural antimicrobials and antioxidants, such as tea polyphenols, grape seed extract, and essential oils, have demonstrated efficacy in extending the shelf life of bream, tilapia, and snakehead fillets [5]. Alginate-chitosan bilayer coatings have also been applied to rainbow trout and ready-to-eat baked fish products [1]. Furthermore, polysaccharides derived from marine macroalgae, including fucoidan and acidic polysaccharides from *Scagassum*, have shown preservative effects on fish fillets through antimicrobial and antioxidant mechanisms [1,4]. For golden pompano specifically, an acidic polysaccharide (SCP-B) isolated from *Scagassum* significantly inhibited *Shewanella putrefaciens* and extended the shelf life of cold-stored fillets by at least 2 days [7]. Gelatin-chitosan composite coatings represent another emerging strategy, though the hydrophilic nature of gelatin necessitates modification to improve water vapor barrier properties [1]. The application of this technology relies on two key attributes of the coating materials: the film-forming capacity of the polysaccharide matrix and the bioactive functionality provided by incorporated phytochemicals. As a naturally abundant polysaccharide, Ch has been extensively adopted as a film-forming substrate in food preservation systems, owing to its excellent biocompatibility, biodegradability, and inherent broad-spectrum antibacterial activity [2,3,15,16]. The latter property arises from the positively charged amino groups along its molecular chain, which interact with negatively charged components on microbial cell surfaces, disrupting membrane integrity and inhibiting bacterial growth [2,3]. Beyond its antimicrobial effects, Ch also serves as an effective carrier for co-delivering additional active substances, offering a versatile platform for developing multifunctional food coatings [15,16].

Although previous studies have explored the performance of various plant polyphenol–chitosan composite films, systematic reports on the physicochemical properties, structural characterization, and application effects of Chitosan–*Sargassum fusiforme* polyphenol edible films (CSFFs) in the refrigerated preservation of aquatic products are still lacking. In particular, the mechanisms by which the intermolecular interactions between phlorotannins (brown algal polyphenols) in *S. fusiforme* and Ch affect the microstructure, mechanical properties, barrier properties, and antibacterial/antioxidant activities of the composite film, as well as the regulatory mechanisms of this composite film on quality attributes such as pH, total bacterial count (TBC), total volatile basic nitrogen (TVB-N), weight loss, texture properties, and color during actual aquatic product storage, remain to be elucidated.

In this study, SP were compounded with Ch to prepare an edible composite film. The chemical composition, physicochemical properties, structural characteristics, and antibacterial/antioxidant activities of the film were systematically characterized, and its quality preservation effects and shelf-life extension capability during refrigerated storage (4 °C) of golden pompano fillets were further evaluated. This study aims to establish a new technological approach to upgrade the utilization of *S. fusiforme* resources, shifting from conventional dietary use towards functional applications in food packaging, thereby enhancing CSFF performance in preserving golden pompano fillets and improving their economic added value.

## 2. Materials and Methods

### 2.1. Materials and Chemicals

Mature *S. fusiforme* was collected from Shangjie Town, Minhou County, Fuzhou City, Fujian Province, China, and purchased from a local agricultural market. Species identity was confirmed by morphological identification based on thallus characteristics (holdfast, stipe, and frond morphology) followed by molecular authentication through ITS or rbcL sequence analysis, with voucher specimens deposited at China Agricultural University. Freshly farmed golden pompano was obtained from a local aquatic product market in Beijing, China, and transported to the laboratory on crushed ice. The fish were obtained as whole, ungutted specimens with an average weight of (500.00 ± 25.00) g and body length of (25.00 ± 2.00) cm. The fish were slaughtered at the fishing site by ice-water immersion before being transported to the laboratory in insulated boxes with crushed ice.Ch (degree of deacetylation > 90%, molecular weight 400 kDa) was purchased from Beijing Solarbio Science & Technology Co., Ltd. (Beijing, China). Pathogenic bacterial strains, including *Vibrio parahaemolyticus* (VP), *Listeria monocytogenes* (LMO), *Escherichia coli* (EC), *Staphylococcus aureus* (SA), *Salmonella enterica* (SE), and *Pseudomonas aeruginosa* (PAG), were obtained from the China Medical Microbiology Culture Collection Center (Beijing, China). The corresponding strain numbers are as follows: *V. parahaemolyticus* CMCC(B) 21617; *L. monocytogenes* CMCC(B) 54004; *E. coli* CMCC(B) 44102; *S. aureus* CMCC(B) 26003; *S. enterica* CMCC(B) 50335; and *P. aeruginosa* CMCC(B) 10104. Deionized water (resistivity 18.2 MΩ·cm) was purified using a Milli-Q system (Merck Millipore, Burlington, MA, USA). Analytical-grade chemicals and reagents were purchased from Sinopharm Chemical Reagent Co., Ltd. (Shanghai, China).

### 2.2. Extraction, Purification, and LC-MS/MS Analysis of SP

The extraction and purification of SP were performed according to the method described by Wang et al. [10]. Identification and characterization of polyphenolic compounds were carried out using a liquid chromatography-tandem mass spectrometry (LC-MS/MS) system. The analysis was conducted on an Agilent 1290 Infinity II liquid chromatograph coupled to an Agilent 6470 triple quadrupole mass spectrometer (Santa Clara, CA, USA), equipped with an Eclipse Plus C18 column. (2.1 mm × 100 mm, 1.8 μm; Agilent Technologies, Santa Clara, CA, USA). The mobile phase consisted of (A) 0.1% (*v*/*v*) formic acid in water and (B) acetonitrile, using a gradient elution program at a flow rate of 0.3 mL/min. The column temperature was maintained at 40 °C, and the injection volume was 2 μL. Mass spectrometric detection was performed in negative electrospray ionization mode, with a scan range of *m*/*z* 100–1500.

### 2.3. Fabrication of SP-Incorporated Composite Films

Composite films based on Ch and containing SP were fabricated via the solution casting approach reported by Liu et al. [1], with minor adjustments. In a typical procedure, 2 g of Ch was dispersed in 100 mL of an aqueous solution composed of 1% (*v*/*v*) acetic acid and 0.5% (*w*/*v*) glycerol, the latter acting as a plasticizing agent. The resulting mixture was subjected to magnetic stirring at ambient temperature until complete dissolution of Ch was achieved, followed by centrifugation at 10,000× *g* for 15 min at 4 °C to eliminate insoluble residues. Thereafter, SP was introduced into the Ch solution at varying mass fractions (0.5%, 1.0%, 1.5%, and 2.0%, *w*/*v*) together with 0.2% (*w*/*v*) Tween 80 functioning as an emulsifying agent, and the system was magnetically agitated for 30 min. The obtained film-forming solution (FFS) was then degassed in a mild ultrasonic bath. Subsequently, the solution was poured into polytetrafluoroethylene molds and subjected to drying at 40 °C for 24 h. Upon completion of drying, the resulting films were detached and equilibrated under ambient conditions at 50% relative humidity for 72 h. Five distinct film formulations were established in this work, namely pristine chitosan film (Ch), Ch film containing 0.5% SP (Ch-SP 0.5), 1.0% SP (Ch-SP 1.0), 1.5% SP (Ch-SP 1.5), and 2.0% SP (Ch-SP 2.0).

### 2.4. Characterization of Physicochemical Properties of the Films

#### 2.4.1. Thickness and Mechanical Attributes

Rectangular strips were excised from the films, and thickness was determined with a CD-30C vernier caliper (Mitutoyo Corporation, Kawasaki, Japan) possessing a precision of 0.001 mm. Five random locations were measured per specimen, and the mean was taken. For tensile strength (TS) and elongation at break (EB) evaluation, film samples measuring 5 cm × 5 cm were prepared. Testing was conducted on a CT3 texture analyzer (Brookfield Engineering Laboratories, Inc., Middleboro, MA, USA) with an initial grip distance of 50 mm and a crosshead velocity of 1 mm/s. Each reported value represents the average of five independent measurements. TS and EB were derived using the following equations:

(1) $$\mathrm{TS}\;(\mathrm{MPa})\;=\;\frac{P}{b\;\times \;d\;}$$

(2) $$\mathrm{EB}\;(\%)=\frac{L-L0}{L0\;}\;\times \;100$$

where *P* denotes the maximum tensile force (N), b represents the film thickness (mm), d corresponds to the film width (mm), *L* is the film length at rupture (mm), and *L*0 is the initial film length (mm).

#### 2.4.2. Moisture Content (MC) and WS

MC and WS were assessed following the protocol of Zhao et al. [16] with minor adaptations. In brief, 5 cm × 5 cm film specimens were oven-dried at 105 °C until a constant weight (w1) was attained, after which they were submerged in distilled water for 1 h under continuous magnetic stirring. The remaining insoluble film material was then dried at 105 °C to a constant weight (w2). MC and WS were computed as follows:

(3) $$\mathrm{MC}\;(\%)\;=\;\frac{w-w1}{w\;}\;\times \;100$$

(4) $$\mathrm{WS}\;(\%)=\frac{w1-w2}{w\;1}\;\times \;100$$

#### 2.4.3. Water Vapor Permeability (WVP)

WVP was evaluated by means of the gravimetric cup technique as described by Song et al. [2], with slight modifications. Briefly, films were trimmed into circular pieces of 5 cm diameter. Glass bottles (4.0 cm diameter) holding 10 mL of deionized water were covered with the film samples and transferred to a desiccator containing anhydrous calcium chloride at ambient temperature. The weight of each bottle was recorded at 12 h intervals, and WVP was obtained from the following expression:

(5) $$\mathrm{WVP}\;=\;\frac{\Delta m\;\times \;d}{A\;\times \;t\;\times \;\Delta p}\;\times \;100$$

where Δm (g) is the weight change in the glass bottle, d (cm) is the film thickness, t (s) is the permeation time, A (cm^2^) is the effective area of the film, and ΔP (kPa) is the water vapor pressure difference across the film.

#### 2.4.4. Film Color

Film color was assessed with a portable colorimeter (CR-10 Plus, Konica Minolta, Tokyo, Japan) following calibration against a white standard plate. The parameters recorded were lightness (L*), redness/greenness (a*), and yellowness/blueness (b*). Additionally, the total color difference (ΔE*) and chroma (C*) were determined as described by Liu et al. [1]:

(6) $$\Delta E*\;=\;\sqrt{{\left(\Delta L*\right)}^{2}+{(\Delta a*)}^{2}+{(\Delta b*)}^{2}}$$

(7) $$C*=\sqrt{{\left(a*\right)}^{2}+{\left(b*\right)}^{2}}$$

where $\Delta L^{*}=L_{\mathrm{standard}}^{*}-L_{\mathrm{sample}}^{*}$, $\Delta a^{*}=a_{\mathrm{standard}}^{*}-a_{\mathrm{sample}}^{*}$, and $\Delta b^{*}=b_{\mathrm{standard}}^{*}-b_{\mathrm{sample}}^{*}$.

#### 2.4.5. Opacity Value

Film opacity was determined on a UV5500 UV-Vis spectrophotometer (Yuanxi Instrument Co., Ltd., Shanghai, China) by measuring the absorbance at 600 nm. The opacity value was derived from the equation reported by Liu et al. [3]:

(8) $$\mathrm{Opacity}\;\mathrm{value}\;=\;\frac{A600}{d}$$

where $A_{600}$ is the absorbance at 600 nm, and d (mm) is the film thickness.

#### 2.4.6. Rheological Properties

The viscosity of the FFS was measured using a HAAKE MARS40 rheometer (Thermo Fisher Scientific, Waltham, MA, USA) equipped with a coaxial cylinder system (outer diameter 30 mm, inner diameter 26 mm) at a constant temperature of 25 °C over a shear rate range of 0–100 s^−1^. Data acquisition and instrument control were performed using HAAKE RheoWin Software (version 4.91, Thermo Fisher Scientific, Karlsruhe, Germany). The obtained flow curves were subsequently processed and analyzed using OriginPro 2021 (OriginLab Corporation, Northampton, MA, USA), in which the shear rate–viscosity data were fitted to the Power Law model to quantify the flow behavior index (n) and consistency coefficient (K).

### 2.5. Structural Characterization of the Films

#### 2.5.1. Scanning Electron Microscopy (SEM)

The films were cut into small pieces, sputter-coated with gold, and observed using an S4800 field emission scanning electron microscope (Hitachi, Tokyo, Japan) at an accelerating voltage of 10.0 kV to examine the surface and cross-sectional microstructures. The acquired SEM images were processed and quantified using ImageJ software (version 1.54f; National Institutes of Health, Bethesda, MD, USA).

#### 2.5.2. Fourier Transform Infrared (FTIR) Spectroscopy

Samples were mixed with KBr powder at a mass ratio of 1:50 and pressed into pellets [3]. FTIR spectra were collected using a Spotlight 400 Frontier infrared spectrometer (PerkinElmer, Waltham, MA, USA) over a scanning range of 4000–400 cm^−1^ at a resolution of 4 cm^−1^ with 64 scans.

#### 2.5.3. Particle Size Distribution (PSD)

The PSD was measured using a Zetasizer Nano ZS90 laser particle size analyzer (Malvern Instruments, Malvern, UK). The FFS was diluted with distilled water to achieve an optimal obscuration range (10–15%) for laser diffraction, and particles ranging from 0–3500 μm were measured. All measurements were performed in triplicate. The PSD, area mean diameter D[3,2] (μm), and volume mean diameter D[4,3] (μm) were recorded. In addition, the $D_{10}$, $D_{50}$, and $D_{90}$ values of the particles in the FFS were recorded.

### 2.6. Bioactive Properties of the Films

#### 2.6.1. Antioxidant Activity

The DPPH and ABTS radical-scavenging activities of SP and the prepared films were measured as previously reported [1] with modifications. DPPH assay: 1.5 mL sample + 1.5 mL DPPH solution, 30 min, ambient temperature, dark, 517 nm. ABTS assay: ABTS^+^• working solution prepared from 3.5 mM ABTS and 1.225 mM potassium persulfate (1:1, *v*/*v*), 14 h in the dark, diluted with phosphate buffer (pH 7.34) to A_734_ = 0.70 ± 0.01; 1.5 mL working solution + 1.5 mL sample, 20 min, room temperature, dark, 734 nm.

#### 2.6.2. Antibacterial Activity

The antibacterial activity of CSFFs was evaluated using the Oxford cup method according to Wei et al. [4] with slight modifications. CSFFs containing 0%, 0.5%, 1.0%, 1.5%, and 2.0% SP were tested against six pathogenic bacterial strains: VP, LMO, EC, SA, SE, and PAE. Bacterial suspensions were prepared in sterile saline and adjusted to a turbidity equivalent to the 0.5 McFarland standard (approximately 1 × 10^8^ CFU/mL), after which a 100 μL aliquot was evenly spread onto nutrient agar (NA) plates. Oxford cups (6 mm inner diameter) were then placed onto the inoculated agar surface, and 100 μL of Ch, CSFFs, or FFS was added to each cup, with an equal volume of distilled water serving as the blank control. After incubation at 37 °C for 24 h, the diameters of the inhibition zones were measured using a vernier caliper. The antibacterial activity was classified based on the inhibition zone diameter as follows: ≤6 mm, no antibacterial activity; >6 mm and ≤10 mm, low sensitivity; >10 mm and ≤15 mm, moderate sensitivity; >15 mm, high sensitivity.

### 2.7. Chitosan-SP Composite Film Coating for Golden Pompano Fillets

Fresh golden pompano was eviscerated and thoroughly rinsed with sterile water. The fish were then cut into fillets with uniform shape and thickness. Subsequently, the obtained fillets were uniformly coated with chitosan solutions incorporated with different mass fractions of SP (0.0%, 0.5%, 1.0%, 1.5%, and 2.0%, *w*/*v*). All the coated fillet samples were stored at 4 °C until their quality indicators reached the inedible limit.

### 2.8. Variations in Physicochemical Quality Indicators

#### 2.8.1. pH Measurement

The pH of golden pompano fillets was monitored at 3-day intervals following a previously described procedure. In brief, 3 g of minced golden pompano fillet was combined with 30 mL of distilled water, homogenized for 30 min, and subsequently centrifuged at 10,000× *g* for 5 min at 4 °C. The supernatant was then harvested, and pH was recorded with an FE20 digital pH meter (Mettler-Toledo Technology (China) Co., Ltd., Shanghai, China).

#### 2.8.2. TVB-N Measurement

The TVB-N content of golden pompano fillets was quantified following the protocol of Liu et al. [1] with minor adaptations. Specifically, 10 g of fish fillet was combined with 100 mL of distilled water and homogenized. Following centrifugation, the supernatant was collected, and 1 g of MgO was introduced for alkalization and distillation for 5 min on a DK20 Kjeldahl distillation apparatus (VELP Scientifica, Usmate Velate, Italy). The distillate was trapped in an absorption flask containing boric acid solution (20 g/L) and subsequently titrated with 0.01 M HCl, employing a few drops of methylene blue (0.1%, *w*/*v*) and methyl red (0.1%, *w*/*v*) as indicators. Results were expressed as mg N per 100 g of fish fillet.

### 2.9. Determination of TBC in Fish Fillets

The TBC of fish fillets, serving as a marker of microbial deterioration, was enumerated via the agar plate counting technique as described by Yang et al. [5]. Fish fillets were placed into sterile stomacher bags containing 0.85% (*w*/*v*) sterile saline. Following homogenization for 5 min, 10-fold serial dilutions were prepared using sterile saline. A 0.1 mL aliquot of each dilution was evenly spread onto plate count agar and incubated at 37 °C for 24 h. Results were reported as log colony-forming units per gram (log CFU/g). Specifically, 2.5 g of fish fillet tissue was combined with 22.5 mL of sterile saline (0.85%, *w*/*v*) and homogenized at 9000 rpm for 2 min. A 1 mL aliquot of the homogenate was diluted with 9 mL of sterile saline to yield a 10-fold diluted sample. Subsequently, 1 mL of the most recent diluted homogenate was transferred to sterile agar plates. Once the agar had solidified, the plates were inverted and incubated at 30 °C for 72 h. Following enumeration, results were expressed as log CFU/g of fish fillet tissue.

### 2.10. Determination of Weight Loss of Golden Pompano Fillets

Weight loss was indicated by the change in fish fillet weight throughout storage. Fillet weight was recorded at 3-day intervals, and weight loss was computed using the following equation:

(9) $$\mathrm{Weight}\;\mathrm{loss}\;(\%)=\frac{w_{0}-w_{i}}{w_{0}}\times 100$$

where $w_{0}$ is the initial weight of the fish fillet, and $w_{i}$ is the weight of the stored fish fillet.

### 2.11. Evaluation of Colour and Texture Properties of Fish Fillets

#### 2.11.1. Color Difference Measurement of Fish Fillets

Color variation in fish fillets during storage was periodically assessed with a CR-400 colorimeter (Konica Minolta, Japan). Following calibration against a standard plate, measurements were conducted at three locations on the fillet surface. The L* (0-black, 100-white), a* (positive-red, negative-green), and b* (positive-yellow, negative-blue) values were recorded. The ΔE* of the fish fillets was calculated following a previously described method using the equation below:

(10) $$\Delta E*\;=\;\sqrt{{\left(L-L_{0}\right)}^{2}+{\left(a-a_{0}\right)}^{2}+{\left(b-b_{0}\right)}^{2}}$$

where L, a, and b represent the color values of the treated fish fillets during storage, and $L_{0}$, $a_{0}$, and $b_{0}$ represent the initial color values of fresh fish fillets.

#### 2.11.2. Springiness and Hardness

The hardness and springiness of fish fillets were assessed on a CT3 texture analyzer (Brookfield Engineering Laboratories, Inc., Middleboro, MA, USA) following the method of Ye et al. [17]. The instrument was fitted with a spherical probe of 5 mm diameter, and the test speed was set at 1 mm/s. Measurements were performed at three random locations on the fish fillet at 3-day intervals, with three replicate measurements at each point, and the mean value was calculated.

### 2.12. Statistical Analysis

All experiments were performed in triplicate. The results were expressed as mean ± standard deviation. One-way analysis of variance (ANOVA) was performed using SPSS 20.0 statistical software to evaluate the differences between factors and levels. Duncan’s multiple range test was used to identify significant differences between groups, with the significance level set at p<0.05.

## 3. Results and Discussion

### 3.1. Chemical Composition and Antioxidant Activity of SP

The chemical composition of SP mainly consisted of 13 small-molecule compounds, including 11 phlorotannins (predominantly fuhalol-type) and two flavonoids. This compositional profile was consistent with previous findings on SP reported by Wang et al. [10]. As shown in Figure S1A,B, the DPPH and ABTS radical scavenging activities of SP increased in a dose-dependent manner with increasing polyphenol concentration. Notably, at a concentration of 50 μg/mL, the ABTS radical scavenging capacity of SP was comparable to that of vitamin C. This result was attributed to the abundant phenolic hydroxyl groups in SP, particularly the pyrogallol moieties unique to fuhalol-type phlorotannins, which can efficiently neutralize ABTS cationic radicals through HAT or SET mechanisms. Furthermore, the possible synergistic antioxidant effects among multiple small-molecule compounds may compensate for the insufficient activity of individual components. Similar concentration-dependent antioxidant behavior has also been observed in studies on coconut peel polyphenols [18].

### 3.2. Thickness and Mechanical Properties of CSFFs

**Figure 1:**
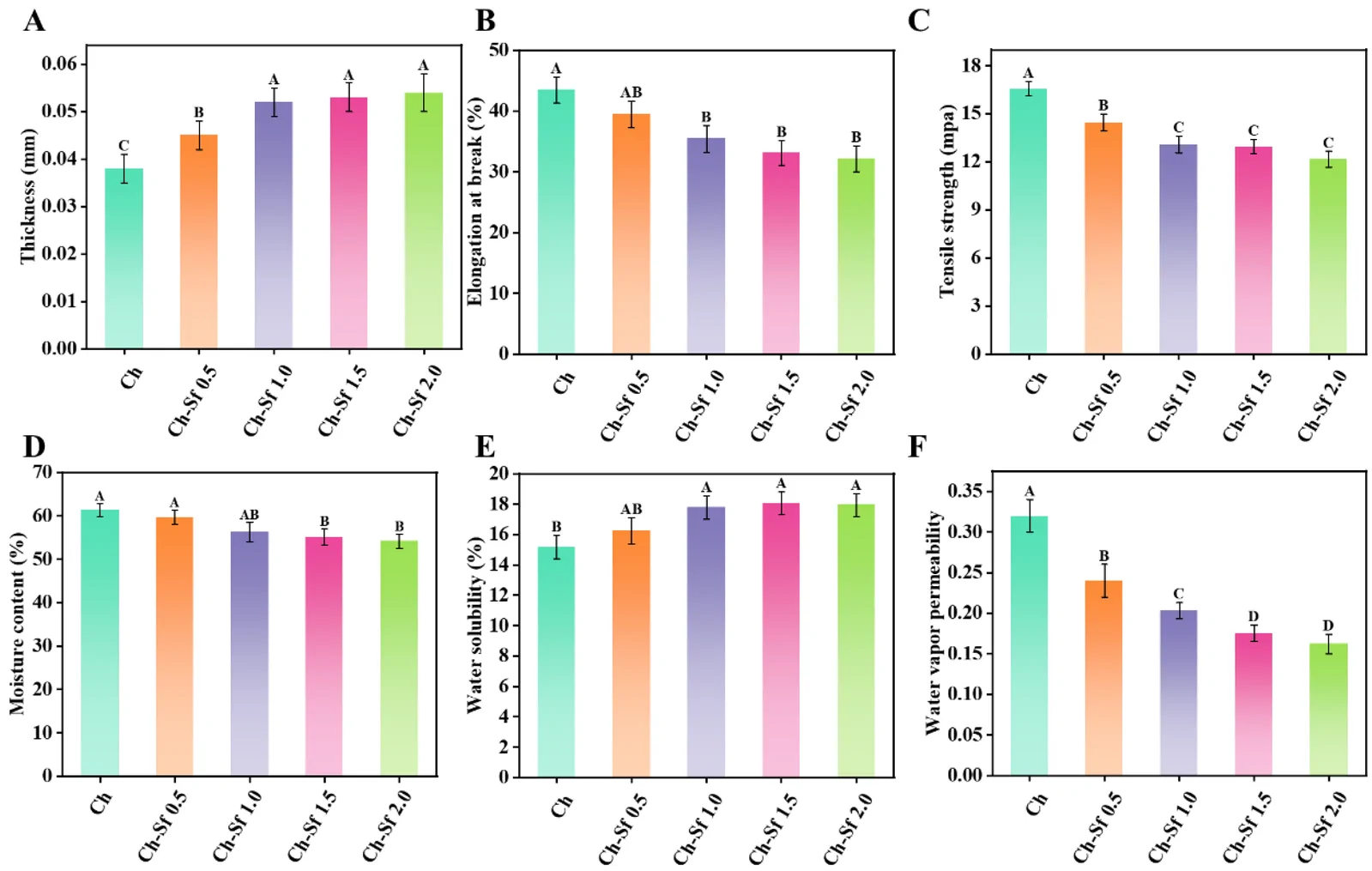
Physicochemical properties of Ch film and composite films loaded with CSFFs at concentrations of 0.5%, 1.0%, 1.5%, and 2.0%: (**A**) Thickness; (**B**) EB; (**C**) TS; (**D**) MC; (**E**) WS; (**F**) WVP. Different letters denote significant differences at *p* < 0.05.

The thicknesses of Ch films and CSFFs are shown in Figure 1A. Compared with pure Ch films, the incorporation of SP significantly affected film thickness. This was mainly attributed to the abundant phenolic hydroxyl groups and benzene ring structures in SP, which formed strong intermolecular hydrogen bonds and hydrophobic interactions with the amino and hydroxyl groups on Ch molecular chains. These interactions led to changes in the ordered arrangement of polymer segments and the density of interchain crosslinking, subsequently affecting the rheological behavior of the FFS and the packing mode of molecular chains during solid-state film formation, ultimately manifesting as significant alterations in film thickness [6]. De Carli et al. [9] investigated the addition of propolis extract to Ch-based biodegradable active films and found that the incorporation of polyphenolic components significantly increased film thickness compared with pure Ch films. They attributed this phenomenon to the densification of the polymer network structure and increased solid content induced by polyphenol–Ch intermolecular hydrogen bonding [9].

The mechanical properties of the films, expressed as TS and EB, are critical parameters determining the ability of food packaging to withstand external forces during storage and logistics. As shown in Figure 1B,C, the incorporation of SP resulted in a progressive reduction in both TS and EB. Compared with the pure Ch group, when the SP proportion increased to 1.0%, 1.5% and 2.0%, TS decreased by approximately 21.01%, 21.73% and 26.56%, respectively, while EB decreased by approximately 18.49%, 23.79% and 26.08%, respectively. These deteriorations reflect a complex, concentration-dependent interplay of multiple intermolecular mechanisms involving hydrogen bonding, electrostatic interactions, crosslinking effects, and microstructural alterations.

At lower SP concentrations (≤0.5%), the abundant phenolic hydroxyl groups of SP may form new hydrogen bonds with the hydroxyl and amino groups of chitosan, creating additional crosslinking nodes within the polymer network. This partial crosslinking could theoretically increase chain rigidity, yet this effect appears to be outweighed by the concurrent disruption of the original inter- and intra-molecular hydrogen bonding network among chitosan chains [19]. The competitive hydrogen-bonding between SP and Ch, rather than reinforcing the matrix, likely weakens the cohesive energy density of the film, as similarly reported for Ch films incorporated with Zanthoxylum bungeanum leaf polyphenols [20] and tea polyphenol nanoparticles [21].

At higher SP concentrations (>1.0%), the situation is further complicated by polyphenol self-aggregation through intermolecular hydrogen bonding and hydrophobic interactions. Such aggregates act as stress-concentration points and introduce local structural defects within the film matrix, as observed by Li et al. [21] for unevenly dispersed polyphenol nanoparticles. Additionally, the dense hydroxyl-rich architecture of SP may engage in electrostatic interactions; specifically, the polyphenolic hydroxyl groups can form charge-transfer complexes or weak ionic associations with protonated amino groups (–NH_3_^+^) of chitosan under the acidic film-casting conditions, potentially altering the charge distribution along the polymer backbone and affecting chain mobility [19].

The combined effect of these molecular-scale interactions is manifested in the film microstructure. The disruption of the original hydrogen-bonding network, coupled with the formation of polyphenol aggregates and the introduction of electrostatic perturbations, reduces the overall structural homogeneity of the composite films. This compromised network integrity, characterized by increased free volume and reduced chain entanglements, directly accounts for the observed declines in both TS and EB [19,20]. Furthermore, the crosslinking-versus-plasticisation duality of polyphenols should be noted: while moderate crosslinking could reinforce the matrix, the overwhelming hydrogen-bond competition and aggregate-induced defect formation at higher SP loadings dominate the mechanical outcome, shifting the balance towards structural weakening. Taken together, these mechanistically distinct but interconnected effects- competitive hydrogen bonding, electrostatic interactions, polyphenol self-aggregation, and microstructural heterogeneity-collectively explain the concentration-dependent reduction in mechanical performance of the SP-Ch composite film matrix.

### 3.3. MC, WS, and WVP of CSFFs

Figure 1D shows the effects of different addition levels of SP on the MC of the prepared composite films. Pure Ch films exhibited the highest MC value (61.34 ± 1.43%). With the incorporation of SP, MC values showed a decreasing trend, and when the addition ratios were 1.5% and 2.0%, MC decreased to 55.12 ± 1.84% and 54.12 ± 1.64%, respectively. Given that SP (and Tween 80) are non-volatile at the drying temperature (40 °C), their incorporation inevitably increases the total solid fraction within the film matrix, which partially contributes to the apparent decline in the calculated MC percentage. However, this physical dilution effect alone is insufficient to fully account for the observed reduction. The predominant mechanism is mainly attributed to the hydrophobic benzene ring structures in SP and the intramolecular/intermolecular hydrogen bonding competition formed with Ch molecules, which collectively reduce the effective density of free amino and hydroxyl groups available for water molecule binding on the Ch chains, thereby intrinsically decreasing the water-absorbing capacity of the film network [6]. Dai et al. [22] also reported a significant decrease in MC after incorporating epigallocatechin gallate (EGCG) into the Ch matrix, explaining that the hydrophobic benzene ring structure of EGCG and the formation of polyphenol–Ch hydrogen bonds collectively reduced the effective density of hydrophilic functional groups in the polymer matrix, thereby weakening the binding and retention capacity of the film for water molecules.

WS is an important indicator for evaluating the biodegradability of films. In this study, the WS of pure Ch films was 15.16 ± 0.78%, while after the addition of 1.0%, 1.5%, and 2.0% SP, the WS of the composite films increased to 17.78 ± 0.77%, 18.05 ± 0.75%, and 17.95 ± 0.75%, respectively (Figure 1E). Clearly, the incorporation of SP significantly increased the WS of the films, and this trend became more pronounced when the addition ratio exceeded 1.0%. The main mechanism was that the introduction of polyphenols disrupted the original dense hydrogen bonding network between Ch molecules, leading to the formation of more free volume and microporous structures in the film matrix, thereby enhancing the penetration and swelling capacity of water molecules [12]. Meanwhile, the hydrophilic hydroxyl groups of some polyphenols also increased the surface wettability of the film [23]. Similarly, Westlake et al. [24] observed that with the introduction of green tea polyphenols, the WS of vanillin cross-linked Ch composite films was higher than that of pure Ch films, attributing this phenomenon to the reduced crosslinking density and loosened polymer network structure caused by hydrogen bonding competition between polyphenols and Ch [24].

WVP serves as an indicator of a film’s capacity to impede the transmission of water vapor and even oxygen from the surrounding environment, and represents a critical factor governing food shelf life. As illustrated in Figure 1F, the WVP of pristine Ch films was 0.32 ± 0.02 mm/kPa·h·m^2^. Upon increasing the SP incorporation ratio to 1.5% and 2.0%, the WVP of the composite films declined to 0.17 ± 0.01 and 0.16 ± 0.01 mm/kPa·h·m^2^, respectively. Such a reduction may be explained by the following mechanisms: the ordered arrangement of the hydrophobic aromatic ring skeletons of SP within the Ch matrix lengthened the diffusion pathway of water vapor molecules [11]; concurrently, hydrogen-bonding crosslinking between polyphenols and Ch diminished the free volume of the film, while the inherently low hydrophilicity of polyphenols weakened the film’s capacity to adsorb and transmit water molecules [11]. Qu et al. [25] likewise reported that the WVP of tuna skin collagen–Ch composite films containing pomegranate polyphenols was lower than that of the control group, and noted that the interaction between the hydrophobic domains of polyphenols and the polymer matrix served as the structural foundation for establishing a dense water vapor barrier layer [25]. In addition, Cazón et al. [26], in their systematic investigation of the WVP of Ch films, explicitly demonstrated that incorporating hydrophobic compounds (e.g., lipids or polyphenols) could lower WVP via a dual mechanism: on one hand, by diminishing the surface adsorption capacity of the film toward water molecules, and on the other hand, by elevating the tortuosity of the diffusion pathway of water molecules within the film matrix. A reduction in WVP signifies an improvement in water vapor barrier performance, which is typically attained by introducing hydrophobic lipid compounds through mechanisms that diminish water adsorption capacity and augment the tortuosity factor for water molecule transfer and diffusion within the film matrix [26].

### 3.4. Color and Opacity of CSFFs

The optical properties of the films, including colour and opacity, are important factors affecting the appearance of coated foods and consumer acceptance. As shown in Table 1, the opacity of pure Ch films was 3.89 ± 0.15. As the SP addition ratio increased from 0.5% to 2.0%, the opacity of the composite films increased to 5.13 ± 0.22, 6.12 ± 0.24, 7.88 ± 0.26 and 7.43 ± 0.27 A/mm, respectively. This increase is mainly attributable to intermolecular interactions between SP and the Ch matrix, which promote structural densification and create additional light-scattering sites [27]. Nxumalo et al. [28] similarly reported that plant extract incorporation reduces film transparency, attributing this to both the chromogenic nature of the extracts and polyphenol-induced matrix densification. In addition to the intrinsic pigmentation of the algae extract, two reaction pathways during film casting may further contribute to the development of brown polymers and the concomitant colour changes. First, phenolic compounds in SP can undergo oxidative polymerization, particularly under the aerobic and slightly alkaline conditions often encountered during film-forming solution preparation and drying, generating high-molecular-weight brown pigments such as quinones and their coupling products [27,28]. Second, the film casting process involves heating and partial dehydration, conditions that can promote Maillard reactions between the reducing ends of polysaccharides (e.g., chitosan) and amino groups, as well as between Maillard precursors present in the extract and the Ch matrix, yielding brown melanoidin-like polymers [27,28]. Both pathways are therefore likely to act in concert with the inherent colour of the extract, collectively accounting for the progressive darkening and increased opacity observed in the composite films.

Notably, the incorporation of SP also substantially altered the colour parameters of the composite films, as summarized in Table 1. The L* value decreased with increasing SP content, particularly beyond 1.0% addition, indicating reduced brightness, while both a* and b* values increased, resulting in marked rises in ΔE* and C*. These colour changes are first and foremost attributable to the intrinsic pigmentation of the SP extract itself. Because the algal material was not subjected to decolourisation pretreatment during extraction or film preparation, the naturally occurring chromogenic compounds present in SP are directly incorporated into the film matrix, inevitably imparting a visible colour shift. In addition to this primary pigment-related effect, the oxidative polymerisation of phenolic compounds abundant in SP during film formation, alongside potential Maillard-type reactions between polyphenols and Ch, may have further contributed to the generation of quinone-derived coloured species or brown polymers, thereby intensifying the overall colouration [29]. Overall, the increased opacity and the observed colour characteristics of the composite films may help protect packaged foods from discolouration and oxidative damage induced by visible or ultraviolet light, potentially reducing the loss of photosensitive nutrients [1].

### 3.5. Rheological Viscosity of CSFFs

**Figure 2:**
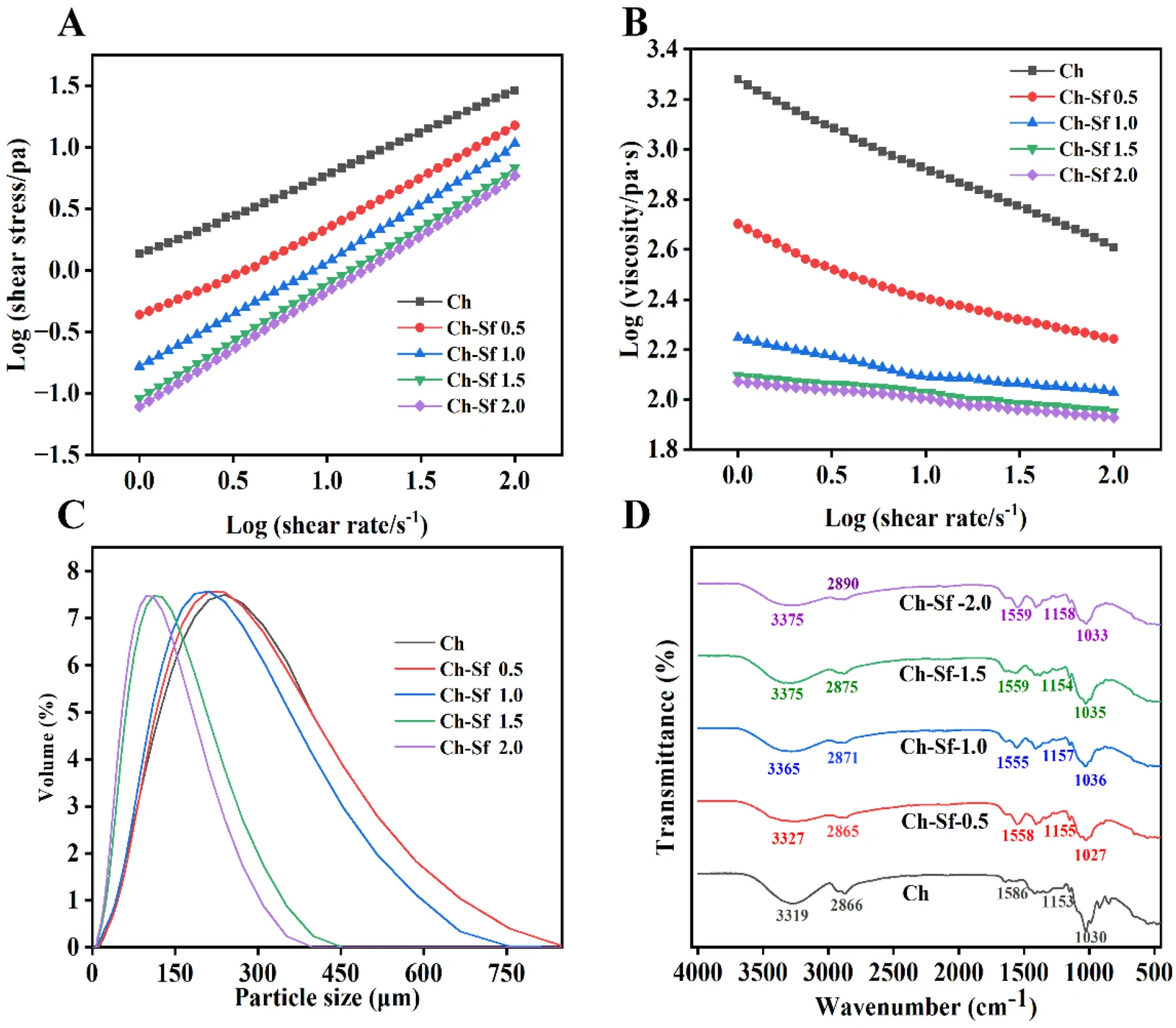
Rheological properties, particle size distribution, and chemical structure of Ch film and composite films incorporated with CSFFs at concentrations of 0.5%, 1.0%, 1.5%, and 2.0%. (**A**) Rheological viscosity curves; (**B**) Flow curves; (**C**) FTIR spectra; (**D**) Particle size distribution of the FFS.

The viscosity of the FFS is a critical determinant of the physical and mechanical characteristics of the resulting films. The influence of SP incorporation on the shear rheological behavior of the FFS is presented in Figure 2A,B. As the shear rate increased, the apparent viscosity of all FFS formulations declined progressively, whereas the shear stress rose continuously, displaying typical shear-thinning characteristics. The slope of the shear stress–shear rate curves revealed that the pristine Ch solution possessed the highest apparent viscosity. Moreover, once the SP addition ratio surpassed 1% (*w*/*v*), the downward trend in viscosity tended to plateau. This phenomenon was primarily ascribed to non-covalent interactions formed between the phenolic hydroxyl moieties of SP and the Ch molecular chains, which partially compromised the original three-dimensional physical crosslinking network among polymer chains, consequently diminishing flow resistance. Comparable findings have likewise been documented by [30].

### 3.6. Structural Characterization of CSFFs

#### 3.6.1. SEM Analysis

**Figure 3:**
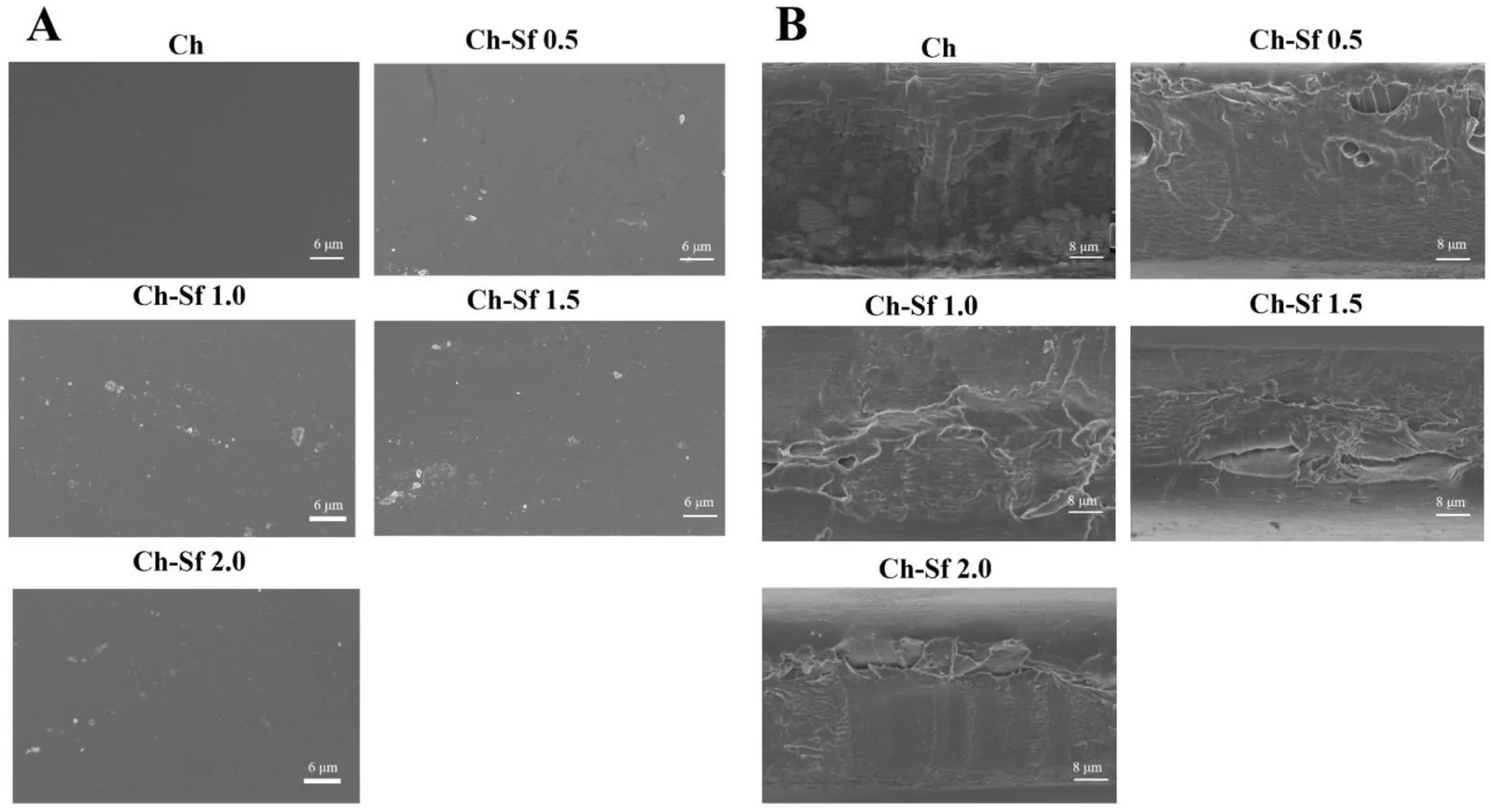
SEM micrographs of Ch film and composite films incorporated with CSFFs at concentrations of 0.5%, 1.0%, 1.5%, and 2.0%. (**A**) Fracture surface morphology; (**B**) Cross-sectional morphology.

The physical properties of the films are determined by their microstructure. The effect of SP incorporation on the microstructure of the composite films is shown in Figure 3. The results indicated that the microstructure of the composite films changed. Pure Ch films exhibited a smooth, flat, and relatively uniform surface without obvious pores or cracks (Figure 3A). This was mainly due to the formation of strong hydrogen bonding networks and intermolecular interactions between the Ch molecular chains, which promoted an ordered and compact packing structure during film formation. With increasing concentration of SP, the film surface gradually became more heterogeneous, characterized by increased roughness and the appearance of uniformly distributed micropores, indicating that SP was effectively incorporated into the Ch matrix. This phenomenon may be attributed to the formation of non-covalent bonds between the phenolic hydroxyl groups of polyphenols and the amino/hydroxyl groups of Ch, which disrupted the ordered arrangement of the original polymer chains and induced local phase separation during solvent evaporation [31]. Furthermore, with increasing amounts of bound SP, the porous structure of the film cross-section became more pronounced (Figure 3B), possibly due to the traction generated by the contraction of the Ch network during solvent evaporation [32].

#### 3.6.2. FTIR Spectroscopy Analysis

FTIR analysis was used to evaluate the functional groups of Ch and the intermolecular interactions between components in the CSFFs, as these interactions directly affect the physical and mechanical properties of the films. As shown in Figure 2C, although the FTIR spectra of the composite films exhibited characteristic peaks similar to those of Ch, the intensities and positions of the main peaks varied with the addition ratio of SP. All films showed a characteristic absorption band near 3350 cm^−1^ and an absorption peak near 2880 cm^−1^, corresponding to O–H stretching vibrations and C–H stretching vibrations of methylene/methyl groups, respectively. In addition, the peaks near 1550 cm^−1^ and 1150 cm^−1^ were attributed to the amide II band (N–H bending vibration) and the asymmetric stretching vibration of glycosidic bonds (C–O–C), respectively. The peak near 1030 cm^−1^ was associated with the C–OH stretching vibration in the Ch backbone. Zhou et al. [33] investigated Ch/bacterial cellulose composite films incorporated with tea polyphenol nanoliposomes and found that the introduction of tea polyphenols caused a clear red shift (from 3410 cm^−1^ to 3385 cm^−1^) and broadening of the O–H/N–H stretching vibration peak near 3350 cm^−1^, accompanied by the appearance of a new peak near 1650 cm^−1^ attributed to the C=C stretching vibration of polyphenol benzene rings [33]. They attributed these changes to the formation of strong intermolecular hydrogen bonds between the abundant phenolic hydroxyl groups of tea polyphenols and the –NH_2_ and –OH groups on Ch molecular chains, thereby demonstrating the existence of non-covalent interactions between polyphenols and the polymer matrix.

#### 3.6.3. Particle Size Distribution

As shown in Figure 2D, both pure Ch and CSFFs exhibited unimodal and polydisperse PSD patterns. With an increasing proportion of SP, the PSD of the FFS showed a clear leftward shift and narrowing of the peak shape. This indicated that upon the introduction of SP, strong hydrogen bonding and hydrophobic interactions formed between the polyphenols and Ch molecules, inhibiting the aggregation and entanglement of Ch molecular chains and reducing the size of aggregates in the solution system. The most pronounced PSD shift occurred after the addition of 1.5% SP, suggesting that at this concentration, the formation of polyphenol–Ch complexes reached a critical assembly equilibrium, maximizing the homogenization of particle distribution in the solution. Furthermore, as shown in Table 2, the pure Ch solution had the largest particle size, with volume mean diameter D[4,3] and surface area mean diameter D[3,2] of 316.45 ± 23.44 μm and 182.33 ± 11.23 μm, respectively. The addition of SP affected the PSD of the mixed FFS. Consistent with the PSD shift, the D[4,3], $D_{10}$, $D_{50}$, and $D_{90}$ values of the solutions containing SP all decreased (Table 2), reaching the lowest values at an addition ratio of 2.0% SP. Moreover, as the addition ratio of SP increased from 0.5% to 2.0%, the D[3,2] values of the particles in the mixed FFS decreased, indicating an increase in particle surface area. The decrease in particle size indicated that the FFS became more uniformly dispersed, with improved homogeneity and stability of the system, favoring the formation of composite film materials with denser structures and more uniform distribution of functional activity, highlighting their potential value from an application perspective [34]. Chen et al. [35] also found in their study on the preparation of tea polyphenol–Ch nanoparticles that the introduction of tea polyphenols caused a significant leftward shift in the PSD and a reduction in the polydispersity index of the Ch-based system, attributing this to the intermolecular hydrogen bonds formed between the phenolic hydroxyl groups of tea polyphenols and the amino/hydroxyl groups of Ch, which effectively inhibited the excessive entanglement of Ch molecular chains and promoted the formation of more uniform and stable composite nanoparticles [35].

### 3.7. Antioxidant Activity of CSFFs

The DPPH and ABTS radical scavenging activities of the FFS were determined to evaluate their in vitro antioxidant capacity following SP incorporation. Compared with pure Ch, the CSFFs exhibited superior radical-scavenging activity. As shown in Figure S2, the antioxidant capacity of the CSFFs increased in a concentration-dependent manner, reaching a maximum for both assays at an SP incorporation level of 15%. This enhancement is plausibly attributable to the abundant phlorotannins in SP, which are known to serve as efficient hydrogen-atom donors capable of neutralizing DPPH and ABTS cationic radicals through free-radical chain-termination reactions [7]. Consistent with this observation, Wang et al. [36] reported that the incorporation of tea polyphenols into a Ch-based system increased DPPH and ABTS scavenging rates from 12.8% and 8.5% to 86.4% and 79.2%, respectively, which they attributed to the abundant phenolic hydroxyl groups acting as potent hydrogen donors. In contrast, pure Ch films exhibited only modest radical-scavenging activity, consistent with the limited number of free amino groups and the absence of abundant phenolic hydroxyl structures that are characteristic of polyphenolic substances, which limits the availability of hydrogen atoms or electrons to neutralize free radicals; additionally, dense intra- and intermolecular hydrogen bonding within the Ch matrix may shield residual active sites, further restricting its direct radical-scavenging capacity [37].

Importantly, while the in vitro radical-scavenging activity correlates positively with SP concentration, establishing a direct quantitative link between this chemical antioxidant capacity and the preservation of fish quality during refrigerated storage remains methodologically challenging within the current experimental framework. We acknowledge that the present study did not evaluate lipid oxidation markers such as PV or TBARS, which would provide more direct evidence for oxidative stability in the fish matrix. Nevertheless, we contend that the observed preservation effects, specifically the significant suppression of microbial proliferation (TVC) and protein degradation (TVB-N) in SP-coated samples, are collectively consistent with a multifunctional mechanism in which antioxidant phenolics may contribute indirectly to shelf-life extension. This could occur through mitigating oxidative stress derived from both endogenous enzymatic activity and microbial metabolism, thereby retarding the formation of secondary spoilage products. However, we explicitly recognize that this proposed pathway remains inferential rather than definitively proven; the specific contribution of radical-scavenging to oxidative stability, particularly regarding lipid-derived rancidity, requires dedicated investigation using PV and TBARS analyses in future studies before any causal relationship can be firmly established.

### 3.8. Antibacterial Activity of CSFFs

The antibacterial activity of the Ch solution and CSFFs against two Gram-positive bacteria (SA and LMO) and four Gram-negative bacteria (PA, EC, SE, and VP) was evaluated using the Oxford cup method. As shown in Table 3, compared with Ch alone, SP incorporation conferred enhanced inhibition against all six tested pathogens, indicating that SP serve as the primary bioactive component. This activity is plausibly attributable to the phlorotannin-rich composition of SP. The phenolic hydroxyl groups of these compounds can interact with proteins and lipids in the bacterial cell membrane, potentially compromising membrane integrity and increasing permeability. Additionally, phenolic-mediated redox cycling may induce intracellular oxidative stress, as previously proposed for plant polyphenols [7,38].

Regarding the differential susceptibility between Gram-positive and Gram-negative bacteria, a two-way analysis of variance (ANOVA) with bacterial Gram type and SP concentration as factors, followed by Tukey’s post hoc test, was performed to compare the mean inhibition zone diameters across groups (Table 3). The results showed that Gram-positive bacteria (SA and LMO) exhibited significantly larger inhibition zones than Gram-negative bacteria (PA, EC, SE, and VP) across all SP concentrations (*p* < 0.05). This differential response is consistent with the known structural differences between bacterial taxa and has been widely reported for plant polyphenols and phlorotannin-rich extracts, which generally show higher activity against Gram-positive strains [39,40]. The greater sensitivity of Gram-positive bacteria may be because their cell envelope lacks an outer membrane barrier; although their peptidoglycan layer is thicker, it is relatively porous, allowing small polyphenolic molecules to more easily penetrate and access the cytoplasmic membrane. In contrast, the outer membrane of Gram-negative bacteria is composed of tightly packed lipopolysaccharides, forming an efficient charge and hydrophobic barrier that impedes the transmembrane diffusion of such compounds [41]. Moreover, after the SP incorporation ratio exceeded 1.5%, the antibacterial activity reached a plateau, suggesting saturation of available binding sites on the bacterial surface. Higher polyphenol concentrations may reduce the effective monomeric fraction owing to intermolecular aggregation or precipitation [42]. Excessive polyphenols can form self-aggregates via hydrogen bonding and hydrophobic interactions, decreasing the number of active molecules that can enter the membrane and bind to targets; concurrently, extensive crosslinking with bacterial extracellular polymeric substances may physically shield some membrane surface binding sites.

Beyond the direct action of SP, a possible synergistic contribution from the chitosan matrix itself warrants consideration. Chitosan, a cationic polysaccharide, is known to exert antibacterial effects through electrostatic interactions with negatively charged bacterial surfaces, leading to local membrane disorganization [8]. Within the composite films, this membrane-perturbing effect may facilitate the access of co-released SP-derived phenolics to the lipid bilayer, potentially lowering the threshold concentration required for membrane disruption [6,8]. Conversely, the dense crosslinked Ch–SP network formed during film preparation could modulate the release kinetics of active phenolics, thereby influencing the observed bacteriostatic outcomes. Nevertheless, deconvoluting the precise additive versus synergistic contributions of each component, as well as the exact release–action dynamics, lies beyond the scope of the current diffusion-based assay and merits dedicated investigation in future studies [6,8].

### 3.9. Application Effects of CSFFs in the Preservation of Golden Pompano

#### 3.9.1. Effect of CSFFs on Ph Changes of Golden Pompano Fillets

**Figure 4:**
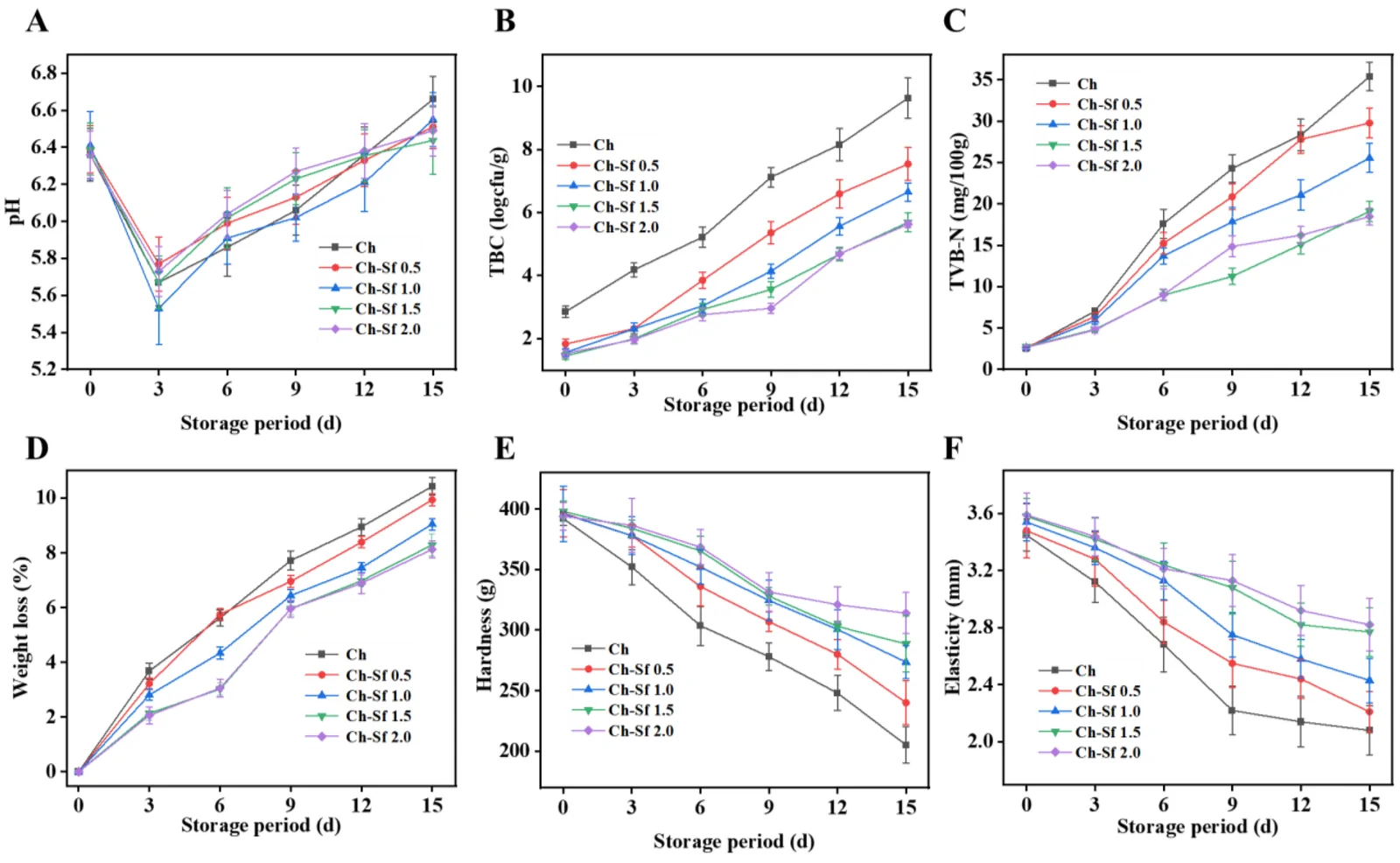
Quality changes in golden pompano fillets during refrigerated storage following various treatments. Physicochemical and microbiological properties: (**A**) pH value; (**B**) TBC; (**C**) TVB-N; (**D**) Weight loss (%). Textural properties: (**E**) Hardness; (**F**) Elasticity.

pH is closely related to the sensory quality of fish and has been used as a feasible reference for determining freshness. A pH value above 6.7 is generally considered an indicator of spoilage. As shown in Figure 4A, during the first 0–3 days of refrigerated storage, the pH of all treatment groups decreased. This was mainly due to the anaerobic glycolysis of glycogen in the muscle postmortem, producing lactic acid, and the rapid decomposition of ATP into ADP and AMP, further generating phosphate groups and H^+^, leading to a rapid short-term accumulation of hydrogen ions in the tissue. Among them, the group with 1.0% SP showed the largest decrease, dropping by approximately 0.88 (or 2.31%). Furthermore, as the storage period extended at 4 °C, the pH increased. This was attributed to the metabolism of nitrogen-containing compounds by endogenous enzymes and psychrophilic spoilage bacteria, producing alkaline volatile substances such as ammonia, trimethylamine, putrescine, and cadaverine, which gradually neutralized and reversed the initially acidified microenvironment [41,42]. Notably, compared with the Ch group, the treatment groups with added SP showed a slower pH increase, with pH values remaining below 6.7 after 15 days. This was because the phlorotannins abundant in SP, on the one hand, reduced the production of alkaline metabolites by destroying bacterial cell membrane integrity and inhibiting the proliferation and decarboxylase/deaminase activities of spoilage bacteria. On the other hand, their phenolic hydroxyl structures could effectively scavenge free radicals and chelate metal ions, delaying the oxidative degradation of unsaturated fatty acids and proteins in the fish, thereby avoiding microenvironmental acidification or pH fluctuation caused by the accumulation of oxidation products and maintaining the relative stability of pH during the middle and late stages of storage.

#### 3.9.2. Effect of CSFFs on TBC Changes of Golden Pompano Fillets

TBC is an indispensable parameter for evaluating the safety and edibility of aquatic products. The critical value for acceptable quality is generally considered to be 7 log CFU/g. The changes in TBC of fish fillets after different treatments and during refrigerated storage are shown in Figure 4B. Compared with Ch, the growth rate of TBC in the treatment groups with added SP was well inhibited. This was attributed to the phlorotannins in SP, which can effectively destroy bacterial cell membrane structures, interfere with the microbial electron transport chain and enzyme activity systems, and simultaneously induce intracellular ROS accumulation, thereby inhibiting the proliferation of psychrophilic spoilage bacteria. Particularly with the addition of 1.5% and 2.0% SP, after 15 days of refrigerated storage, the TBC values were 5.69 ± 0.31 and 5.63 ± 0.11 log CFU/g, respectively, which were below 7 log CFU/g. This was because high concentrations of polyphenols not only provided stronger antibacterial activity but also formed a denser composite film structure with the Ch matrix, continuously releasing active phenolic components through a sustained release effect, thereby maintaining a long-lasting inhibitory effect on target spoilage bacteria during prolonged refrigerated storage [43]. Similarly, Beigizadeh and Bazargani-Gilani [44] investigated Ch/polyvinyl alcohol composite films loaded with beetroot peel extract for the preservation of trout fillets and found that the addition of plant extract delayed the increase in TBC of fish fillets during refrigerated storage, attributing this antibacterial effect to the disruption of bacterial cell membrane permeability and the inhibition of microbial metabolic activity by the active phenolic components in the extract [44].

#### 3.9.3. Effect of CSFFs on TVB-N Changes of Golden Pompano Fillets

TVB-N is considered an important indicator for evaluating the spoilage of aquatic products, mainly caused by nitrogen-containing compounds generated from the degradation of proteins and nucleic acids due to the activities of proteolytic enzymes and endogenous enzymes. A level of 25 mg N/100 g is considered the maximum acceptable limit for fish fillets and aquatic products. As shown in Figure 4C, compared with Ch, Ch with SP effectively inhibited the formation of TVB-N. This was because the phlorotannin components in SP acted simultaneously on both endogenous enzymes and microorganism-derived proteases, reducing the generation of alkaline nitrogen-containing volatiles such as ammonia, trimethylamine, and putrescine at the source. Notably, after 15 days of refrigeration at 4 °C, compared with the TVB-N of Ch (35.37 ± 1.74 mg N/100 g), the TVB-N values for 1.5% and 2.0% SP were 19.10 ± 1.23 and 18.45 ± 1.04 mg N/100 g, respectively, which were below 25 mg N/100 g. This was because higher concentrations of SP (≥1.5%) provided stronger initial antibacterial activity and enzyme inhibition in the Ch film matrix, and also continuously released effective concentrations of phenolic active components through sustained release during 15 days of refrigerated storage, thereby maintaining long-term inhibition of TVB-N generation. Zamani et al. [45] found similar results when applying Ch nano-coating loaded with green cumin extract for the preservation of rainbow trout.

#### 3.9.4. Evaluation of Weight Loss of Golden Pompano Fillets by CSFFs

The weight loss of golden pompano fillets is shown in Figure 4D, and all groups showed an increasing trend during refrigerated storage. This indicated a continuous decline in the water-holding capacity of the fish during storage, with intracellular water continuously exuding and accumulating in the interstices or on the surface of the muscle tissue, which may be related to factors such as protein oxidative degradation, disruption of myofibrillar structure, and loss of cell membrane integrity [13]. The Ch group showed the most significant weight loss, possibly because although pure Ch film could form a certain physical barrier, its water resistance was limited, and the hydrophilic amino and hydroxyl groups on the Ch molecular chains tended to adsorb free water, creating a locally moist microenvironment at the film–meat interface, accelerating the evaporation and exudation of water from the fish surface [46]. Furthermore, compared with the Ch group, the fish fillets treated with CSFFs showed significantly lower values as storage progressed. This observation could be attributed to the formation of a dense hydrogen bonding network between SP molecules and Ch, enhancing the hydrophobicity and barrier properties of the composite film and reducing the migration of water from the fish to the environment [47]. Yu et al. [15] also observed in their study on water-soluble Ch loaded with pomegranate peel extract as a multifunctional bioactive coating for fish fillet preservation that the weight loss of fish fillets with added pomegranate peel extract was lower than that of the pure Ch coating treatment group during refrigerated storage.

#### 3.9.5. Texture Profile Analysis

Texture is a key factor affecting quality evaluation and consumer acceptance. As shown in Figure 4E, the hardness of golden pompano fillets showed varying degrees of decline during storage. The Ch group exhibited the fastest softening rate, decreasing from an initial value of approximately 391 ± 5.44 N to 205 ± 15.11 N on day 15. This was attributed to the limited ability of the pure Ch coating to block oxygen and microorganisms: on the one hand, residual oxygen promoted the oxidative degradation of myofibrillar proteins, disrupting the crosslinking network that maintains muscle structure [48]; on the other hand, the proliferation of spoilage microorganisms released extracellular proteases and collagenases, hydrolyzing cytoskeletal proteins and collagen in the connective tissue, leading to sarcomere loosening and Z-line degradation, thereby accelerating the softening of the fish fillets [49]. However, compared with the Ch group, the CSFF coating effectively prevented the decrease in hardness throughout the storage period. This was due to the multiple protective effects of phlorotannins in SP: first, their strong antioxidant activity could scavenge free radicals and chelate metal ions, inhibiting the oxidative crosslinking failure and degradation of myosin and actin, thereby protecting the integrity of myofibrils [50]; and second, polyphenols could inhibit the activities of microbial proteases and host endogenous proteases, slowing down the decomposition of key structural proteins [8]. In addition, the non-covalent binding formed between polyphenols and proteins may stabilize the three-dimensional network structure of proteins to some extent [51]. Springiness or elasticity, another factor determining texture characteristics, showed the same trend as hardness (Figure 4F). The decrease in hardness and springiness was caused by protein degradation, myofibrillar damage, reduced intercellular adhesion, and water loss, which is usually caused by endogenous proteolytic enzymes and partly due to microbial proliferation [52].

#### 3.9.6. Color Measurement of Golden Pompano Fillets by CSFFs

Color is an important indicator of fish fillet freshness and quality. As shown in Table 4, the Ch group showed the fastest decline in $L^{*}$ and $a^{*}$ values throughout the storage period. Until the end of storage, the $L^{*}$ values of the CSFFs were significantly (p<0.05) higher than those of the control group. Reddening of fish fillets during refrigerated storage is more acceptable to consumers. The changes in $a^{*}$ values among different treatments were relatively stable, indicating that the CSFF treatment did not cause significant red pigment degradation or abnormal oxidative discoloration of myoglobin on the fish fillet surface, which is consistent with its potential to maintain meat color stability. With extended storage time, the $L^{*}$ values decreased, indicating a reduction in the brightness of the fish fillets, which may be attributed to weakened light scattering due to surface moisture evaporation, turbid appearance caused by the accumulation of microbial metabolites, and surface microstructure roughening due to oxidative aggregation of myofibrillar proteins [53]. The $b^{*}$ values of the fish fillets gradually increased during storage, with the 1.5% and 2.0% treatments showing the best effect in delaying the increase in $b^{*}$ values, followed by the 1.0% and 0.5% groups. This was due to the antibacterial and antioxidant effects of SP, inhibiting microbial growth and delaying the discoloration and deterioration of the fish fillets. During storage, severe color differences generally indicate quality deterioration, which is mainly caused by the synergistic effects of multiple factors, including the Maillard reaction between carbonyl compounds generated from lipid oxidation and the amino groups of free amino acids or proteins, the oxidation of myoglobin to metmyoglobin, the accumulation of pigments and biogenic amines produced by microbial metabolism, and structural color changes induced by surface dehydration [14]. Similarly, Zhao et al. [16] found in their study on edible composite films based on Ch nanoparticles for packaging fresh red sea bream fillets that the decrease in $L^{*}$ and $a^{*}$ The values of fish fillets in the active component-loaded composite film treatment group were slower than those in the pure Ch film treatment group during refrigeration at 4 °C.

## 4. Conclusions

In this study, SP were successfully integrated into a Ch matrix using a solution casting method. The SP, which are rich in phlorotannins and flavonoids, exhibited in vitro free radical scavenging capacity, providing a chemical basis for their functional activity. Increasing the proportion of SP incorporation led to significant alterations in the mechanical properties, WVP, optical characteristics, microstructure and bioactivity of the CSFFs. Moreover, the introduction of SP endowed the CSFFs with synergistic antioxidant and antibacterial activities. Notably, under refrigerated storage at 4 °C, coating with CSFFs substantially delayed the quality deterioration of golden pompano fillets. On the basis of the combined mechanical, barrier, optical and bioactivity results, the optimal SP incorporation ratio was identified as 1.0–2.0%, at which the laboratory-scale CSFFs combine favourable physical barrier properties with potent biological activities. It should be emphasized, however, that this optimum was established under laboratory-scale casting conditions and therefore cannot be directly extrapolated to industrial production. The present study does not assess production scalability, economic feasibility, industrial processing compatibility or long-term shelf stability of the films. Therefore, while these findings support the proof-of-concept potential of this formulation for active food packaging, any claim regarding a readily scalable packaging strategy would be premature and requires dedicated further investigation, particularly with respect to the aforementioned industrial and economic factors.

## Figures and Tables

**Table 1:** Color parameters and optical properties of Ch film and composite films incorporated with CSFFs at concentrations of 0.5%, 1.0%, 1.5%, and 2.0%.

	Ch	Ch-Sf 0.5	Ch-Sf 1.0	Ch-Sf 1.5	Ch-Sf 2.0
L*	92.45 ± 0.56 ^A^	91.32 ± 0.54 ^B^	90.45 ± 0.51 ^B^	88.95 ± 0.46 ^C^	88.45 ± 0.46 ^C^
a*	−3.94 ± 0.11 ^C^	0.98 ± 0.06 ^B^	2.89 ± 0.12 ^A^	2.67 ± 0.15 ^A^	2.97 ± 0.16 ^A^
b*	11.67 ± 0.31 ^A^	13.78 ± 0.33 ^C^	16.78 ± 0.35 ^B^	17.43 ± 0.45 ^AB^	18.21 ± 0.41 ^A^
△*E*	0.00 ± 0.00 ^E^	5.50 ± 0.07 ^D^	8.78 ± 0.18 ^C^	9.45 ± 0.21 ^B^	10.33 ± 0.19 ^A^
C*	12.32 ± 0.26 ^D^	13.81 ± 0.33 ^C^	17.03 ± 0.37 ^B^	17.63 ± 0.47 ^AB^	18.45 ± 0.43 ^A^
Opacity value	3.89 ± 0.15 ^D^	5.13 ± 0.22 ^C^	6.12 ± 0.24 ^B^	7.88 ± 0.26 ^A^	7.43 ± 0.27 ^A^

Different uppercase letters (A–E) within the same row indicate statistically significant differences (*p* < 0.05).

**Table 2:** PSD of Ch solution and FFS incorporated with CSFFs at concentrations of 0.5%, 1.0%, 1.5%, and 2.0%.

	Ch	Ch-Sf 0.5	Ch-Sf 1.0	Ch-Sf 1.5	Ch-Sf 2.0
Surface area (m^2^/kg)	35.67 ± 2.24 ^C^	54.78 ± 2.87 ^B^	82.88 ± 3.89 ^A^	84.18 ± 3.65 ^A^	84.45 ± 3.09 ^A^
*D*[3,2] (μm)	182.33 ± 11.23 ^A^	119.76 ± 10.78 ^B^	78.38 ± 6.45 ^C^	74.77 ± 5.64 ^C^	75.87 ± 5.67 ^C^
*D*[4,3] (μm)	316.45 ± 23.44 ^A^	237.43 ± 18.89 ^B^	211.54 ± 17.67 ^B^	208.56 ± 19.23 ^B^	206.65 ± 16.78 ^B^
*D*_10_ (μm)	95.78 ± 6.78 ^A^	73.67 ± 5.34 ^B^	62.78 ± 4.65 ^C^	58.89 ± 4.31 ^C^	56.89 ± 4.53 ^C^
*D*_50_ (μm)	283.89 ± 21.09 ^A^	206.78 ± 18.78 ^B^	186.34 ± 13.67 ^C^	180.87 ± 13.23 ^C^	178.98 ± 12.57 ^C^
*D*_90_ (μm)	598.23 ± 34.25 ^A^	435.56 ± 32.78 ^B^	408.11 ± 32.18 ^C^	387.67 ± 22.67 ^C^	384.89 ± 28.95 ^C^

Different uppercase letters (A–C) within the same row indicate statistically significant differences (*p* < 0.05).

**Table 3:** Inhibition zone diameters (mm) of Ch solution and FFS incorporated with varying concentrations of CSFFs against Gram-negative (*P. aeruginosa*, *E. coli*, *S. enterica*, *V. parahaemolyticus*) and Gram-positive (*S. aureus*, *L. monocytogenes*) bacteria.

	LOM	SA	PAE	EC	SE	VP
Ch	13.15 ± 0.43 ^C^	14.87 ± 0.65 ^B^	13.87 ± 0.65 ^B^	13.54 ± 0.76 ^B^	11.99 ± 0.75 ^B^	12.54 ± 0.87 ^B^
Ch-Sf 0.5	12.33 ± 0.27 ^D^	15.44 ± 0.47 ^B^	14.65 ± 0.27 ^B^	14.43 ± 0.56 ^B^	12.98 ± 0.57 ^B^	13.76 ± 0.76 ^B^
Ch-Sf 1.0	14.54 ± 0.54 ^B^	16.15 ± 0.77 ^AB^	14.88 ± 0.65 ^AB^	15.55 ± 0.87 ^AB^	13.87 ± 0.87 ^AB^	14.57 ± 0.77 ^A^
Ch-Sf 1.5	15.86 ± 0.58 ^A^	17.65 ± 0.86 ^A^	15.31 ± 0.68 ^A^	15.92 ± 0.57 ^A^	14.12 ± 0.68 ^A^	15.18 ± 0.69 ^A^
Ch-Sf 2.0	16.13 ± 0.66 ^A^	17.33 ± 0.58 ^A^	15.54 ± 0.65 ^A^	16.01 ± 0.52 ^A^	14.55 ± 0.86 ^A^	15.32 ± 0.81 ^A^

Different uppercase letters (A–D) within the same column indicate significant differences in inhibition zone diameters among different treatments against the same bacterial strain (*p* < 0.05).

**Table 4:** Color stability of golden pompano fillets subjected to various preservation treatments during cold storage.

L*	**Ch**	**Ch-Sf 0.5**	**Ch-Sf 1.0**	**Ch-Sf 1.5**	**Ch-Sf 2.0**
0 day	40.32 ± 2.13 ^A^	39.89 ± 1.88 ^A^	41.24 ± 1.98 ^A^	41.78 ± 2.37 ^A^	41.31 ± 2.21 ^A^
3 day	36.25 ± 2.56 ^AB^	37.78 ± 2.09 ^AB^	39.78 ± 2.13 ^AB^	39.97 ± 1.87 ^AB^	39.67 ± 1.99 ^AB^
6 day	32.78 ± 2.14 ^B^	33.91 ± 2.11 ^BC^	35.65 ± 1.25 ^BC^	36.22 ± 2.02 ^BC^	36.78 ± 2.31 ^BC^
9 day	28.67 ± 2.54 ^BC^	31.19 ± 2.06 ^C^	33.42 ± 2.23 ^CD^	34.25 ± 2.18 ^CD^	34.67 ± 1.86 ^CD^
12 day	24.15 ± 1.88 ^CD^	27.68 ± 2.17 ^D^	29.78 ± 1.32 ^D^	31.26 ± 1.89 ^DE^	31.67 ± 1.97 ^DE^
15 day	22.14 ± 1.68 ^D^	24.67 ± 2.67 ^D^	26.87 ± 1.43 ^D^	29.56 ± 1.67 ^E^	29.78 ± 2.41 ^E^
***a*** *	**Ch**	**Ch-Sf 0.5**	**Ch-Sf 1.0**	**Ch-Sf 1.5**	**Ch-Sf 2.0**
0 day	2.77 ± 0.12 ^A^	2.89 ± 0.16 ^A^	2.92 ± 0.19 ^A^	2.94 ± 0.12 ^A^	2.93 ± 0.18 ^A^
3 day	2.08 ± 0.17 ^B^	2.11 ± 0.16 ^B^	2.17 ± 0.18 ^B^	2.16 ± 0.17 ^A^	2.22 ± 0.16 ^B^
6 day	0.42 ± 0.02 ^C^	0.38 ± 0.02 ^C^	0.41 ± 0.02 ^C^	0.43 ± 0.02 ^C^	0.40 ± 0.02 ^C^
9 day	−0.34 ± 0.02 ^D^	−0.30 ± 0.02 ^D^	−0.33 ± 0.02 ^D^	−0.29 ± 0.01 ^D^	−0.27 ± 0.01 ^D^
12 day	−0.42 ± 0.03 ^E^	−0.41 ± 0.03 ^E^	−0.38 ± 0.02 ^E^	−0.37 ± 0.02 ^E^	−0.39 ± 0.02 ^E^
15 day	−0.48 ± 0.02 ^F^	−0.46 ± 0.04 ^F^	−0.45 ± 0.03 ^F^	−0.44 ± 0.02 ^F^	−0.43 ± 0.02 ^F^
***b*** *	**Ch**	**Ch-Sf 0.5**	**Ch-Sf 1.0**	**Ch-Sf 1.5**	**Ch-Sf 2.0**
0 day	1.78 ± 0.11 ^E^	1.64 ± 0.11 ^E^	1.69 ± 0.13 ^F^	1.72 ± 0.11 ^E^	1.73 ± 0.12 ^E^
3 day	3.56 ± 0.14 ^D^	3.28 ± 0.21 ^D^	2.97 ± 0.12 ^E^	2.26 ± 0.18 ^D^	2.21 ± 0.18 ^D^
6 day	5.27 ± 0.32 ^C^	4.58 ± 0.33 ^C^	3.35 ± 0.23 ^D^	2.89 ± 0.12 ^C^	2.78 ± 0.20 ^C^
9 day	5.46 ± 0.37 ^BC^	5.17 ± 0.35 ^BC^	3.96 ± 0.24 ^C^	3.32 ± 0.23 ^B^	3.22 ± 0.21 ^B^
12 day	6.16 ± 0.42 ^AB^	5.52 ± 0.32 ^AB^	4.65 ± 0.35 ^B^	3.93 ± 0.27 ^A^	3.87 ± 0.28 ^A^
15 day	6.93 ± 0.43 ^A^	5.92 ± 0.34 ^A^	5.44 ± 0.43 ^A^	4.33 ± 0.33 ^A^	4.31 ± 0.34 ^A^
**△*E***	**Ch**	**Ch-Sf 0.5**	**Ch-Sf 1.0**	**Ch-Sf 1.5**	**Ch-Sf 2.0**
0 day	0.00 ± 0.00 ^E^	1.41 ± 0.93 ^D^	1.64 ± 1.11 ^E^	2.07 ± 1.55 ^CD^	1.81 ± 1.22 ^D^
3 day	4.56 ± 2.25 ^D^	3.20 ± 1.63 ^D^	2.10 ± 1.11 ^E^	1.74 ± 0.70 ^D^	1.81 ± 0.88 ^D^
6 day	8.64 ± 1.86 ^C^	7.49 ± 1.80 ^C^	5.63 ± 1.11 ^D^	5.11 ± 1.66 ^C^	4.75 ± 1.76 ^C^
9 day	12.63 ± 0.52 ^B^	10.29 ± 1.82 ^BC^	8.08 ± 1.93 ^C^	7.23 ± 1.87 ^B^	6.83 ± 1.59 ^B^
12 day	17.05 ± 1.79 ^A^	13.59 ± 2.02 ^AB^	11.47 ± 1.22 ^B^	9.98 ± 1.74 ^AB^	9.61 ± 1.80 ^AB^
15 day	19.12 ± 1.62 ^A^	16.52 ± 2.53 ^A^	14.36 ± 1.35 ^A^	11.63 ± 1.55 ^A^	11.44 ± 2.23 ^A^

Different uppercase letters (A–F) within the same column indicate statistically significant differences (*p* < 0.05).

## Data Availability

The original contributions presented in this study are included in the article/Supplementary Materials. Further inquiries can be directed to the corresponding authors.

## Supplementary Materials

The following supporting information can be downloaded at: https://www.mdpi.com/article/10.3390/foods15203688/s1, Figure S1: ABTS (A) and DPPH (B) free radical scavenging activity of SP. Different lowercase letters indicate statistically significant differences (*p* < 0.05). Figure S2: Photographs of Ch film and composite films incorporated with CSFFs at concentrations of 0.5%, 1.0%, 1.5%, and 2.0%. Figure S3: DPPH and ABTS radical scavenging activities (%) of Ch solution and FFS incorporated with SCCFs at concentrations of 0.5%, 1.0%, 1.5%, and 2.0%. Different lowercase letters indicate statistically significant differences (*p* < 0.05).
