# Supplementary material for: Chitosan–Sargassum fusiforme Polyphenol Edible Films: From Physicochemical and Bioactive Characterization to Shelf-Life Extension of Refrigerated Golden Pompano Fillets
Source: Foods. 2026 Oct 16;15(20):3688. doi: 10.3390/foods15203688 (PMC13704595; doi:10.3390/foods15203688)
Supplement: Supplementary file 1 [file foods-15-03688-s001.zip › foods-4616873-supplementary.pdf]

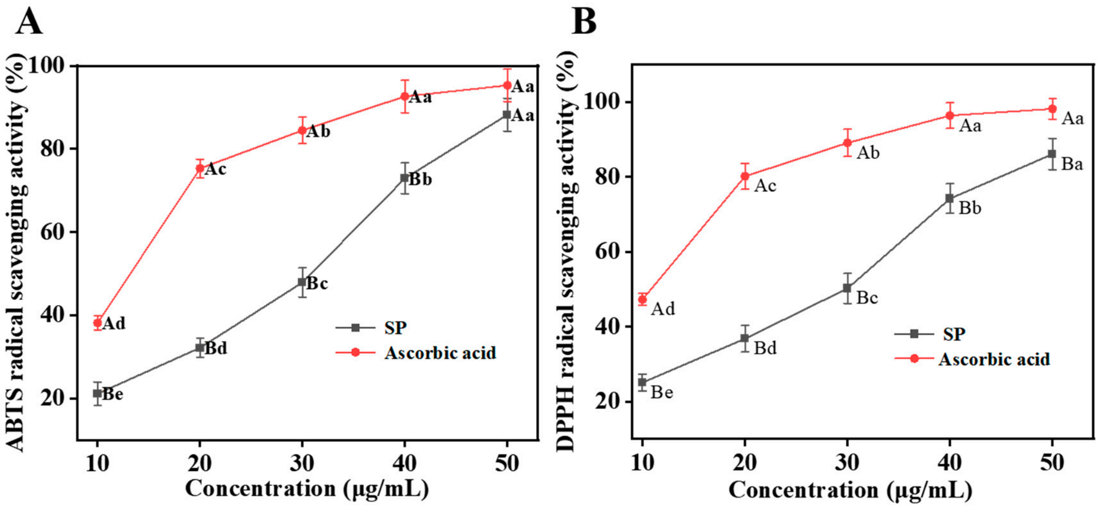

Figure S1: ABTS (A) and DPPH (B) free radical scavenging activity of SP. Different lowercase letters indicate statistically significant differences ($p < 0.05$).

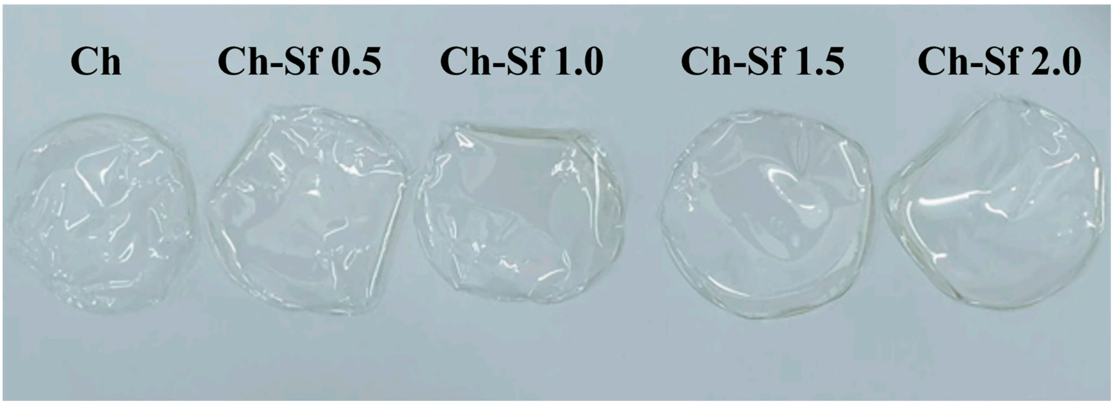

Figure S2: Photographs of Ch film and composite films incorporated with CSFFs at concentrations of 0.5%, 1.0%, 1.5%, and 2.0%.

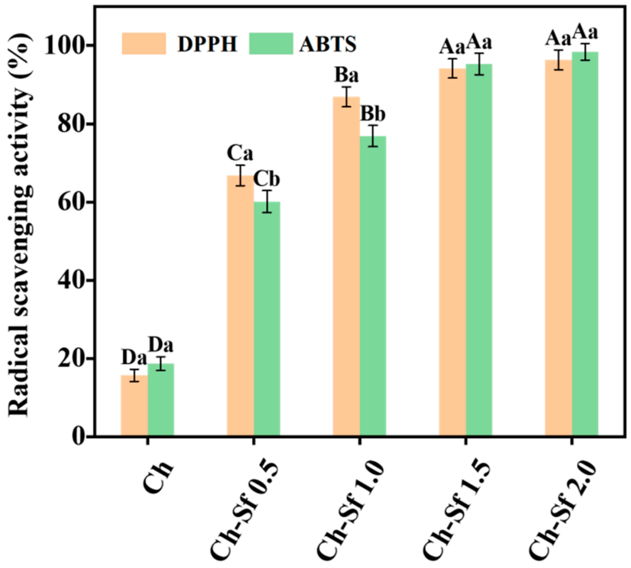

Figure S3: DPPH and ABTS radical scavenging activities (%) of Ch solution and FFS incorporated with SCCFs at concentrations of 0.5%, 1.0%, 1.5%, and 2.0%. Different lowercase letters indicate statistically significant differences ($p < 0.05$).
